# Analysis of nucleotide-binding oligomerization domain proteins in a murine model of pneumococcal meningitis

**DOI:** 10.1186/s12879-014-0648-3

**Published:** 2014-12-02

**Authors:** Xinjie Liu, Qizheng Han, Junhong Leng

**Affiliations:** Department of Pediatrics, Qilu Hospital, Shandong University, No. 107 Wen Hua Xi Road, Jinan, China; Brain Science Research Institute, Shandong University, No. 107 Wen Hua Xi Road, Jinan, China; Department of Respiratory Medicine, Provincial Hospital Affiliated to Shandong University, No. 4 Duan Xing Xi Road, Jinan, China; Department of Ultrasonic Diagnosis, Jinan Maternity and Children Care Hospital, Jian Guo Xiao Jing San Road, Jinan, China

**Keywords:** Meningitis, Gram-positive bacteria, NOD2, Cytokines

## Abstract

**Background:**

The innate immune system recognizes pathogens via its pattern recognition receptors. The objective of this study was to investigate the role of the nucleotide-binding oligomerization domain (NOD) proteins, a family of the novel bacterial pattern recognition receptors, in host responses to the gram-positive bacteria *Streptococcus pneumoniae.*

**Methods:**

Sprague–Dawley rats were infected via intracisternal injections of viable *S. pneumoniae,* and rats in the control group were injected with sterile saline. After infection, real-time PCR was performed to determine the presence of mRNAs encoding NOD1 and NOD2. Quantitative analyses of the NOD1, NOD2 and NF-kB proteins were also performed western blotting following challenge infections with viable *S. pneumoniae.* The TNF-α and IL-6 levels in brain homogenates were evaluated using enzyme-linked immunosorbent assays (ELISAs).

**Results:**

The results revealed up-regulations of the mRNA and protein levels of NOD2 within the CNS of rats with *S. pneumoniae* meningitis. Moreover, the activation of NF-κB in the brain tissues following infection with live *S. pneumoniae* was also significantly increased, which indicates that NOD2 mediated NF-κB activation in experimental pneumococcal meningitis. Similarly, TNF-α and IL-6 levels were increased in the brain following *in vivo S. pneumoniae* administration.

**Conclusions:**

These results suggest that NOD2 is involved in the host response to the gram-positive bacteria *S. pneumoniae* in the CNS and that NOD2 might play an important role in the initiation and/or progression of CNS inflammation associated with pneumococcal meningitis.

**Electronic supplementary material:**

The online version of this article (doi:10.1186/s12879-014-0648-3) contains supplementary material, which is available to authorized users.

## Background

Bacterial meningitis is a serious and often intractable condition that affects the meninges and the brain parenchyma. *Streptococcus pneumoniae* is a common cause of bacterial meningitis, and such infections are associated with neurological sequelae and a high mortality rate [1]-[3]. Once *Streptococcus pneumoniae* have gained access to the central nervous system, the presence of multiplying bacteria within the subarachnoid and ventricular spaces triggers an intense inflammatory host response [4]-[7].

A compelling body of evidence showing that members of the nucleotide-binding oligomerization domain (NOD) family of proteins play a critical role in the immune response following pathogen invasion has accumulated, and at least two members of this family of proteins appear to sense the cytoplasmic microbial pathogen-associated molecular patterns (PAMPs) of the invading microbes and to initiate the activation of the innate immune system, which leads to inflammation [8]-[10]. NOD1, also called caspase recruitment domain (CARD)-4 protein, appears to interact with motifs found in the peptidoglycans of gram-negative bacteria [11],[12]. In contrast, it has been suggested that NOD2 (CARD15) recognizes the bacterial molecules that are produced during the synthesis and/or degradation of peptidoglycan (PGN) [13] and acts a general sensor for most bacteria [7],[8]. Recent studies have demonstrated that microglia and astrocytes express members of the NOD family of proteins that recognize conserved bacterial motifs. To date, the expressions of these novel bacterial pattern recognition receptors have not been established within the central nervous system (CNS) *in vivo*.

In the present study, we provide a demonstration that murine brains constitutively express low levels of NOD2 and show that this expression is up-regulated following the *in vivo* administration of *Streptococcus pneumoniae*. The notion that NOD2 is functional within brains is supported by the observation that this gram-positive CNS pathogen simultaneously elicits increases in the levels of NF-kB protein, which is an essential downstream effector molecule of NOD2-mediated immune responses. Finally, further compelling evidence for the functionality of NOD2 expression in the CNS is provided by a demonstration of the augmentation of inflammatory cytokine production. Taken together, these findings demonstrate that the expression of functional NOD2 proteins in murine brains represents a potentially important mechanism of bacterial infections of the CNS.

## Methods

### Animals and experimental design

This study was performed in strict accordance with the guidelines of the NIH’s Guide for the Care and Use of Laboratory Animals (National Institutes of Health Publication No. 80–23, revised 1996). The protocol was approved by the Animal Care Committee of Shandong University. A total of 48 2-week-old Sprague–Dawley rats were obtained from the Model Animal Research Center of Shandong University (Jinan, PR China). The rats were randomly divided into three infected groups (D1, D2 and D3) and one control group (12 rats in each group). The rats in the infected groups were inoculated with *S. pneumoniae* and sacrificed on day 1 (D1), D2, and D3 after inoculation. The control group was inoculated with sterile saline and sacrificed at 24 h after injection.

### Infecting organism

The serotype 3 strain of *S. pneumoniae* (provided by the National Institute for the Control of Pharmaceutical and Biological Products, Beijing, China) is one of the most common causes of neonatal meningitis and was used in this study. The bacteria was grown on sheep blood agar plates, cultured overnight in VITAL AER broth and incubated overnight at 37°C in air with 5% CO_2_ as described previously by our laboratory. The culture broth was centrifuged, pelleted, resuspended in sterile saline to the desired density and used for intracisternal injection. The inoculum was routinely checked for purity and density with quantitative cultures.

### Intracisternal administration of the bacteria

The operation to induce bacterial meningitis has been previously described by our laboratory [7]. Briefly, infection was induced by direct intracisternal injection of 10 μl of saline containing 1 × 10^6^ of viable *S. pneumoniae* via a 32-gauge needle. Uninfected animals were injected with 10 μl of sterile saline. To confirm the development of bacterial meningitis, CSF was obtained by puncture of the cisterna magna 24 h after inoculation and then cultured, and the brains of the rats were histologically examined. At 1, 2 and 3 days postinfection, the animals were killed, and all brain tissues were removed. The brain tissue was prepared for assessments of NOD1 and NOD2 mRNAs by reverse transcription-PCR (RT-PCR), analyses of the NOD1, NOD2 and NF-kB proteins by western blotting and exploration of the cytokines with specific capture ELISA.

### Reverse transcription-PCR (RT-PCR)

Total RNA from brain tissue was extracted using Trizol reagent following the manufacturer’s instructions. mRNA was isolated by using the Trizol kit and converted to cDNA using the reverse transcriptase. The positive and negative strand PCR primers that were used to amplify the mRNA encoding murine NOD1 (299 bp fragment) were, respectively [13] GTCCTCAACGAGCATGGCGAGACT and AGCTCATCCAGGCCGTCAA. The positive and negative strand primers used to amplify the mRNA encoding murine NOD2 (273 bp fragment) were GCTGCCAATCTTCACGTCGTC and TAAGTACTGAGGAAGCGAGACTGA, respectively. The primers were synthesized by Shanghai Sangon Biological Engineering.

Technology Company Limited. The NOD1 and NOD2 mRNA levels were measured via comparisons to the mRNA level of the housekeeping gene β-actin that were made with a PCR-based method for the quantitation of mRNA that has been described previously in detail by our laboratory [7].

### Western blot analyses of the NOD1, NOD2 and NF-kB protein levels

The quantitative analyses of the NOD1, NOD2 and NF-kB protein levels were accomplished with western blot analyses that were performed essentially as previously described [13]. The rats were killed at the indicated times after infection, and all brain tissue was removed. Protein extraction performed using a Total Protein Extraction Kit and a Nuclear-Cytosol Extraction Kit. The total NOD1 and NOD2 proteins and nuclear NF-κB proteins were prepared. Protein content was measured with a BCA kit according to manufacturer’s instructions. The total protein samples (50 ug) and nuclear protein samples (30 ug) were subjected to electrophoresis on 10% sodium dodecyl sulfate-polyacrylamide gels (SDS/PAGE) and transferred to nitrocellulose membranes. The membranes were blocked with 5% skimmed milk/TPBS (10 mM Tris–HCl, 150 mM NaCl, 0.05% Tween-20) for 2 h at room temperature and then incubated with anti-NOD1 (1:500, Cell Signaling Technology), anti-NOD2 (1: 500, Cell Signaling Technology) and anti-NF-κB P65 antibody (1 : 500, Santa Cruz Biotechnology). The membranes were washed and incubated with secondary antibody for 1 h, and the relative densities of the bands were then analyzed.

### Quantification of cytokine secretion

The TNF-α and IL-6 levels in the brain homogenates were determined using a commercially available ELISA kit according to the directions provided by the manufacturer (Santa Cruz Biotechnology). The cytokine levels in the brain homogenates were normalized to total brain weight and are reported as ng/g of brain tissue.

### Statistical analyses

The data are presented as the means ± the S.D.s, and one-way ANOVA analyses were performed with SPSS13.0 software to compare the differences. A value of p < 0.05 was considered statistically significant.

## Results

### *S. pneumoniae*-induced NOD2 mRNA expression in the brain tissue

**Figure 1:**
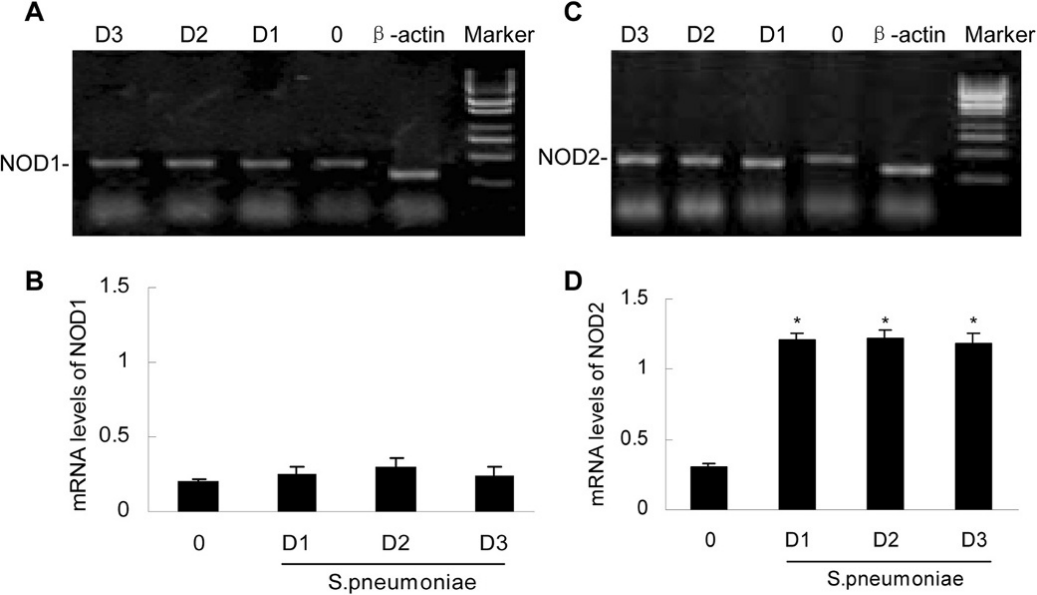
**Electrophoretic determination of the expressions of NOD1 and NOD2 mRNAs (A and C).** Vehicle (0) or *S. pneumoniae* (1 × 10^6^ bacteria) was administered to the rats via intracisternal injection. At days 1 (D1), D2, and D3 postinfection, brain tissue homogenates were isolated for measurements of NOD1 (Panel **A**) and NOD2 (Panel **C**). The expressions of NOD1 and NOD2 mRNAs were quantified by densitometric analyses and normalized to β-actin expression (Panels **B** and **D**). The asterisks indicate statistically significant differences compared to the corresponding uninfected animals (p < 0.05).

To determine whether the murine brain tissue expressed these novel pattern recognition receptors, we assessed the NOD1 and NOD2 mRNA levels in the uninfected and *S. pneumoniae-*infected murine brains. At 1, 2 and 3 days postinfection, RNA was isolated, and real-time PCR was performed to determine the presence of the NOD1 and NOD2 mRNAs. As shown in Figure 1, NOD1 and NOD2 mRNAs were constitutively expressed at very low levels across the entire brains of the uninfected rats. *S. pneumoniae* administration failed to elicit significant increases in NOD1 expression (Figure 1A,B). The *S. pneumoniae*-infected animals exhibited a significant elevation in NOD2 mRNA (Figure 1C,D).

### *S. pneumoniae*-mediated increases in NOD2 protein in the brain tissue following *in vivo*bacterial administration

**Figure 2:**
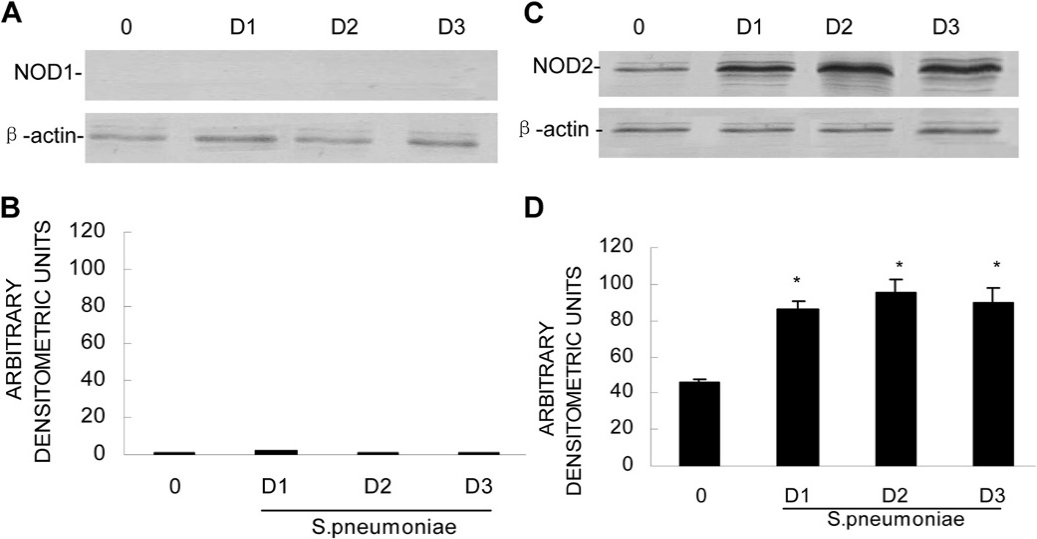
**Western blot analyses of the NOD1 and NOD2 proteins (A and C).** The rats were administered vehicle (0) or were infected with *S. pneumoniae* (1 × 10^6^ bacteria) via intracisternal injection. At days 1 (D1), D2, and D3 postinfection, protein isolates were assayed for the presence of NOD1 (Panel **A**) and NOD2 (Panel **C**). NOD1 and NOD2 protein levels were quantified by densitometric analyses and normalized to β-actin expression (Panels **B** and **D**). The asterisks indicate statistically significant differences compared to the corresponding uninfected animals (p < 0.05).

To determine whether the expression of NOD mRNAs in the brain tissue translated into the expression of the NOD proteins, we performed western blot analyses. As shown in Figure 2, the murine brain tissue constitutively expressed low levels of NOD1 and NOD2 proteins or failed to express these proteins at all. Importantly, *S. pneumoniae* administration resulted in marked increases in NOD2 protein expression. In contrast, the brains expressed little NOD1 protein following bacterial challenge.

### Increased NF-kB expression during acute CNS inflammation following *S. pneumoniae*administration

**Figure 3:**
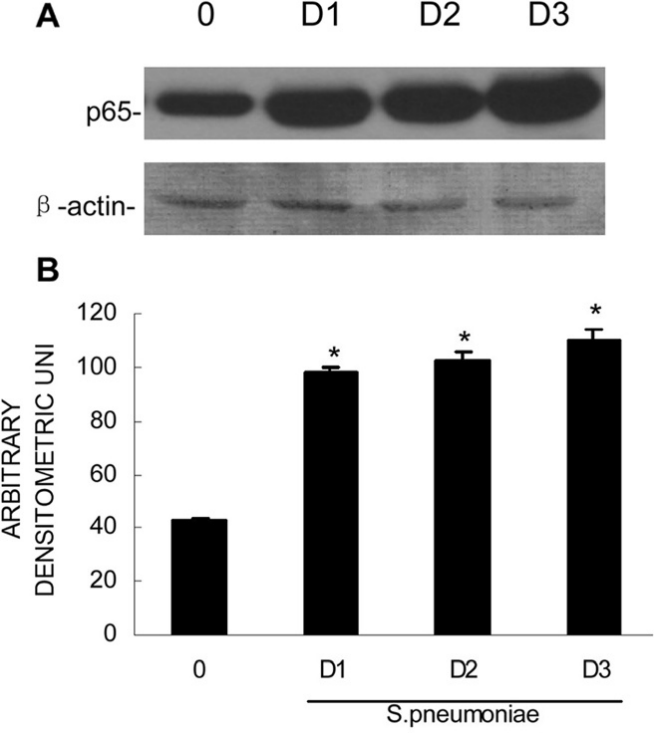
**The activation of NF-kB is increased following infection with** ***S. pneumoniae*** **(A).** Vehicle (0) or *S. pneumoniae* (1 × 10^6^ bacteria) was administered to the rats via intracisternal injection. At days 1 (D1), D2, and D3 postinfection, the brain tissue was isolated, and the nuclear protein was prepared for immunoblot assays of the presence of NF-kB p65 (Panel **A**). The bands were quantified by densitometric analyses and normalized to β-actin expression (Panel **B**). The asterisks indicate statistically significant differences compared to the uninfected animals (p < 0.05).

To determine whether the NOD2 protein is functional in rats, we investigated whether the murine brain tissue expressed NF-kB, which is a critical downstream effector molecule in NOD2-mediated cellular activation. As shown in Figure 3, the brains of the uninfected animals expressed robust levels of this protein. However, the brains of the *S. pneumoniae-*infected rats exhibit significantly higher levels of NF-kB protein expression (Figure 3).

### *S. pneumoniae*-associated increases in inflammatory cytokine levels within the CNS

**Figure 4:**
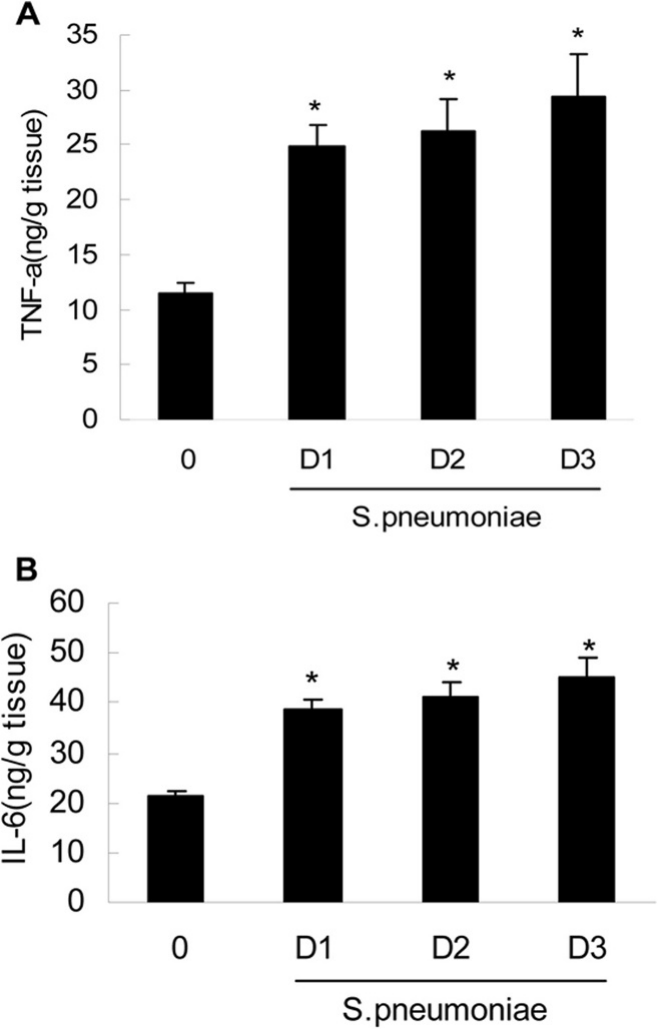
***S. pneumoniae*** **administration elicited elevations in inflammatory cytokine levels within the CNS (A and B).** Infection was induced via direct intracisternal injection of vehicle (0) or *S. pneumoniae* (1 × 10^6^ bacteria). On day 1 (D1), D2, and D3 postinfection, brain tissue homogenates were isolated for assessment of TNF-a (Panel **A**) and IL-6 (Panel **B**) protein expression by specific capture ELISA. The asterisks indicate statistically significant differences compared to the uninfected animals (p < 0.05).

To further evaluate the role of NOD2 protein expression in *S. pneumoniae*-induced neuroinflammation, we assessed the levels of the key inflammatory mediators within the CNS. As shown in Figure 4, *S. pneumoniae* administration significantly increased the levels of the inflammatory cytokines TNF-α (Figure 4 A) and IL-6(Figure 4B) in the CNS.

## Discussion

The innate immune system recognizes pathogens via its pattern recognition receptors. In pneumococcal meningitis, multiple microbial pattern recognition receptors including Toll-like family of the receptors (TLRs) were expressed to perceive bacterial pathogens and to initiate potentially damaging CNS inflammation Klein et al. [14] have recently demonstrated that TLR2 and TLR4 expressed on radioresistant and transplanted BM-derived cells were major cellular sensors of invading *S. pneumoniae* inducing inflammatory responses in murine *S. pneumoniae* meningitis. Mogensen et al. [15] also demonstrated that live *S. pneumoniae* activate the inflammatory response through Toll-like receptors 2, 4, and 9 in species-specific patterns. However, the expression of these receptors does not preclude the involvement of other pattern recognition receptors in the perception of bacterial pathogens. Another subset of pattern recognition receptors are the recently identified nucleotide-binding oligomerization domain (NOD) proteins.

In the present study, we assessed the expression levels of NOD mRNAs and proteins during host recognition of *S. pneumoniae*. We provided evidence for the up-regulations of the expressions of the mRNAs and proteins of these novel pathogen-associated molecular pattern receptors within the CNS following the *in vivo* administration of *S. pneumoniae*. We demonstrated that rat brain tissue constitutively expresses low levels of NOD2 mRNA and detectable levels of NOD2 protein. The expression of NOD2 mRNA and protein were markedly elevated following infection with the gram-positive pathogen *S. pneumoniae*. These observations are consistent with the previously documented ability of murine glial cells to express NOD2 [13],[16],[17], which suggests the involvement of NOD2 proteins in the immune response to *S. pneumoniae* in brain tissue [18]. Currently, the precise mechanisms responsible for this phenomenon have not been determined, although it is possible that the increase in NOD2 expression might result from the recruitment of immune system cells that express NOD2 and not necessarily from an up-regulation of NOD2 expression in the brain cells.

In contrast to our findings related to NOD2, our data indicated that uninfected brain tissues express little or no NOD1, and only a modest level of expression was detectable following infection with the gram-positive bacteria *S. pneumoniae*. This finding agrees with the work of others that has demonstrated that microglia express very low levels of NOD1 mRNA and that exposure of microglia to viable Neisseria meningitis fails to elicit significant increases in NOD1 expression [13]. While the reasons for these findings remain unclear, it is possible that NOD1 only interacts with the naturally occurring peptidoglycan degradation product GlcNAc-MurNAC-L-Ala-gamma-D-Glu-meso-DAP that is found in the peptidoglycans of gram-negative bacteria [11],[12]. More interesting, others [9] have described that intestinal epithelial cells express NOD1 almost exclusively and that gram-negative bacteria are the leading cause of intestinal infections. In contrast, NOD2 appears to function as an intracellular receptor for a minimal motif that is common to all bacterial peptidoglycans [19],[20].

In the present study, we showed that the activation of the pivotal inflammatory transcriptional activator NF-kB in brain tissues following infection with live *S. pneumoniae* was significantly increased. NF-kB binding sites have been identified in the NOD2 promoter [21],[22], and NOD2 has been reported to activate the pivotal transcription factor NF-kB. NOD2 recruits the adaptor molecules RICK (also called RIP2) and CARD9, and the activations of these molecules eventually lead to NF-kB activation [23],[24]. Thus, the present demonstrations of NOD2 expression and inducible NF-kB activation in the brain tissue following the *in vivo* administration of *S. pneumoniae* lend credence to the notion that NOD2 mediates NF-kB activation during host responses to this gram-positive CNS pathogen [3]. Thus, NOD2 might be functional in the host CNS.

A growing body of evidence suggests that once bacteria have gained access to the central nervous system, the pathogens initiate CNS inflammation and that inflammatory mediators are released in this process. Accordingly, others and we have demonstrated the ability of murine glial cells to produce key inflammatory cytokines, including TNF-α and IL-6, in response to bacterial pathogens [25]-[27]. In the present study, we measured the productions of key inflammatory cytokines following *S. pneumoniae* infection *in vivo*. We showed that the infection of rats with *S. pneumoniae* elicited significant increases in inflammatory cytokine production.

Importantly, we observed a simultaneous occurrence of rapid increases in inflammatory mediators, an elevation in NOD2 protein and an increase in NF-k B activation following *in vivo S. pneumoniae* administration. Together, these finding suggest a physiological role of the NOD2 protein in the immune response to pneumococci in the brain tissue.

## Conclusions

Based on the observations of the present study, we demonstrated that NOD2 is involved in host responses to gram-positive bacteria *S. pneumoniae* in the CNS and that NOD2 plays an important role in the initiation and/or progression of the CNS inflammation that is associated with pneumococcal meningitis. Nevertheless, the role of NOD2 in the host response to the gram-positive bacteria *Streptococcus pneumoniae* is currently controversial. NF-kB activation can induce NOD2 up-regulation, and it remains unclear whether NOD2 is activated or plays a role in the inflammatory process that is triggered by *S. pneumonia.* Therefore, further studies are needed to further elucidate these issues. Knowledge of the molecular interactions of pneumococci with brain tissue might lead to improvements in disease outcome.

## Authors’ original submitted files for images

Below are the links to the authors’ original submitted files for images. Authors’ original file for figure 1 Authors’ original file for figure 2 Authors’ original file for figure 3 Authors’ original file for figure 4
