# Supplementary figures and images for: Analysis of nucleotide-binding oligomerization domain proteins in a murine model of pneumococcal meningitis
Source: BMC Infect Dis. 2014 Dec 2;14:648. doi: 10.1186/s12879-014-0648-3 (PMC4256814; doi:10.1186/s12879-014-0648-3)

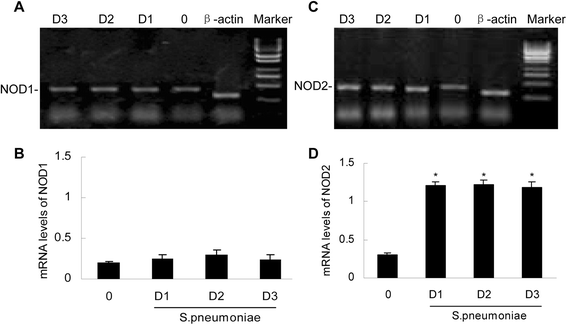

Supplement: Supplementary file 1 — Authors’ original file for figure 1 [file 12879_2014_648_MOESM1_ESM.gif]

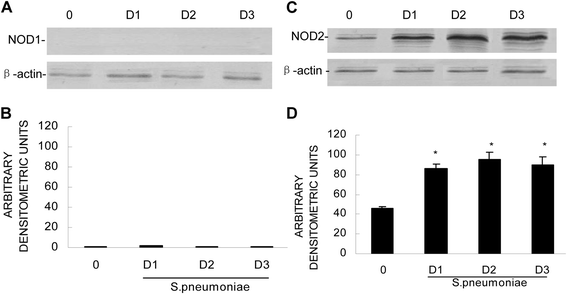

Supplement: Supplementary file 2 — Authors’ original file for figure 2 [file 12879_2014_648_MOESM2_ESM.gif]

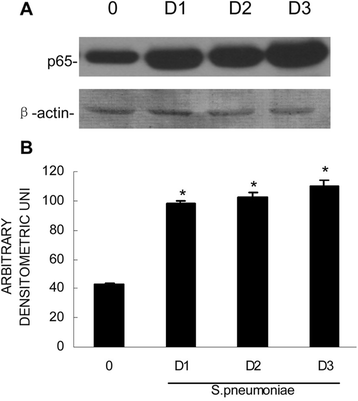

Supplement: Supplementary file 3 — Authors’ original file for figure 3 [file 12879_2014_648_MOESM3_ESM.gif]

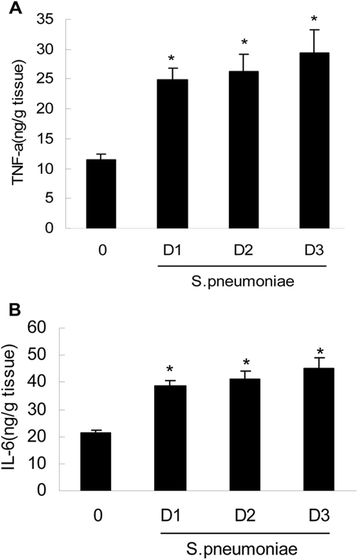

Supplement: Supplementary file 4 — Authors’ original file for figure 4 [file 12879_2014_648_MOESM4_ESM.gif]
